# Iodine Status in Turkish Populations and Exposure to Iodide Uptake Inhibitors

**DOI:** 10.1371/journal.pone.0088206

**Published:** 2014-02-05

**Authors:** Aysel Ozpinar, Fahrettin Kelestimur, Yildiran Songur, Ozge Can, Liza Valentin, Kathleen Caldwell, Ender Arikan, Ibrahim Unsal, Mustafa Serteser, Tamer Inal, Yigit Erdemgil, Abdurrahman Coskun, Nadi Bakirci, Ozlem Sezgin, Ben Blount

**Affiliations:** 1 Department of Medical Biochemistry, School of Medicine, Acibadem University, Istanbul, Turkey; 2 School of Medicine, Erciyes University, Kayseri, Turkey; 3 School of Medicine, Suleyman Demirel University, Isparta, Turkey; 4 Acibadem Hospital, Istanbul, Turkey; 5 Division of Laboratory Sciences, Centers for Disease Control and Prevention, Atlanta, Georgia, United States of America; Kobe University, Japan

## Abstract

Perchlorate, nitrate, and thiocyanate are competitive inhibitors of the sodium iodide symporter of the thyroid membrane. These inhibitors can decrease iodine uptake by the symporter into the thyroid gland and may disrupt thyroid function. This study assesses iodine status and exposure to iodide uptake inhibitors of non-pregnant and non-lactating adult women living in three different cities in Turkey (Istanbul, Isparta and Kayseri). We measured iodine and iodide uptake inhibitors in 24-hr urines collected from study participants (N = 255). All three study populations were mildly iodine deficient, with median urinary iodine (UI) levels of 77.5 µg/L in Istanbul, 58.8 µg/L in Isparta, and 69.8 µg/L in Kayseri. Perchlorate doses were higher in the study population (median 0.13 µg/kg/day), compared with a reference population (median 0.059 µg/kg/day), but lower than the U.S. EPA reference dose (0.7 µg/kg/day). Urinary thiocyanate levels increased with increasing exposure to tobacco smoke, with non-smokers (268 µg/L) significantly lower than light smokers (1110 µg/L), who were significantly lower than heavy smokers (2410 µg/L). This pilot study provides novel data indicating that study participants were moderately iodine deficient and had higher intakes of the iodide uptake inhibitor perchlorate compared with a reference population. Further investigation is needed to characterize the thyroid impact resulting from iodine deficiency coupled with exposure to iodide uptake inhibitors such as perchlorate, thiocyanate and nitrate.

## Introduction

Iodine deficiency disorder is a global health problem affecting 740 million people [1]. The primary reason for iodine deficiency is inadequate dietary iodine intake [1]. Iodine deficiency causes a broad range of health impacts, including increased perinatal mortality, mental retardation, goiter, hypothyroidism, hyperthyroidism, and retarded physical development [2]–[4]. Iodine is a crucial element for maintaining health by enabling production of adequate levels of thyroid hormone. Thyroid hormone synthesis depends upon adequate iodine levels in the thyroid as a result of the pumping action of the transmembrane protein sodium iodide symporter (NIS). NIS transport of iodide ion can be inhibited by environmental chemicals such as perchlorate, thiocyanate, and nitrate. Affinity of perchlorate for the human NIS is 15-fold, 30-fold and 240-fold greater than thiocyanate, iodide and nitrate, respectively [5]. Prolonged inhibition of iodine uptake can lead to decreased thyroid hormone production and ultimately could result in hypothyroidism. Human health effects could result from chronic exposure to NIS inhibitors, particularly in “at risk” populations (pregnant and lactating women, neonates, and children) [6]. Combined chronic effects of perchlorate and thiocyanate exposure may cause decreased iodine transport in both the thyroid and the lactating breast, and possibly lead to reduced thyroid function, hypothyroidism and impaired mental and physical development of offspring.

Turkey has moderate endemic iodine deficiency [1]. In addition, the prevalence of smoking is relatively high in Turkey [7]. According to the “Turkey Demographic and Health Survey 2008”, 22 percent of women currently smoke [7]. The prevalence of smoking among women is gradually (∼10% per 10 years) increasing in Turkey (2008) [7]. Turkey is among the top 10 tobacco-consuming countries in the world [8].

Tobacco smoke contains significant amounts of cyanide that is metabolized in the human body to thiocyanate [SCN^−^]. Thiocyanate can also enter the body through sources such as milk and dairy products. Cigarette smoke exposure can significantly increase thiocyanate concentrations to levels potentially capable of affecting the thyroid gland, especially in populations with low iodine intakes. Knudson et al. (2002) reported that cigarette smokers with low iodine intakes had a higher incidence of goiter compared with smokers with adequate iodine intakes [9]. Thiocyanate has a biological half-life of 1–2 weeks and shares some common physiological properties with iodine [10]. For example, both thiocyanate and iodine are oxidized by peroxidase enzymes.

The combination of low iodine intake, thiocyanate exposure from smoke, and perchlorate exposure may reduce thyroid function in women [11], [12]. The public health strategy to minimize iodine deficiency is salt iodization; in Turkey salt iodization become mandatory in 1998 [13], [14]. Despite these efforts to fortify the population through iodized salt, some populations in Turkey appear to remain iodine deficient [15], [16]. For example, a recent study found low iodine intakes in two cities in Turkey (Burdur [near Isparta] and Kayseri) [16], [17].

Recent studies have also shown that the NIS inhibitors such as perchlorate can decrease iodine uptake by the thyroid [18]–[20]. Perchlorate is used as an oxidizer in solid rocket fuel and it is a component of fireworks, pyrotechnic equipment, and explosives. Perchlorate is also found in Chilean nitrate fertilizers [21]. Perchlorate has been detected in water, beverages, vegetables and dairy products [22]–[30].

Steinmaus et al (2007) showed that thiocyanate and perchlorate exposure are associated with decreased thyroid function in women with low iodine intakes [12]. Recent studies indicated that long-term perchlorate (ClO_4_^−^) exposure, even at low doses, correlates with decreased serum T4 and increased TSH levels in women with low iodine intakes and tobacco smoke exposure [11], [12].

Nitrate is another common NIS inhibitor. Gatseva and Argirova (2007) found that consuming water with high-nitrate levels increases risk for thyroid dysfunction in vulnerable populations [31]. Nitrate intake commonly occurs through diet (e.g. vegetables and cured meats) and drinking water.

The main objective of this pilot study is to characterize exposure to thiocyanate, nitrate and perchlorate in areas of Turkey with differing iodine intakes and potentially elevated levels of iodide uptake inhibitors.

## Materials and Methods

### Recruitment of participants

Volunteer participants were randomly selected from non-pregnant and non-lactating, 18 years of age or older women who were recruited randomly from orthopedics clinic, plastic surgery, physical therapy, psychology, psychiatry, ophthalmology, dermatology, urology, sports medicine, gynecology and neurology clinics. Candidates were screened based on a review of their medical records and a survey that included questions about their lifestyle. These questions assessed topics such as cigarette smoke exposure (non-smoker, light smoker (≤10 cigarettes/day), heavy smoker (>10 cigarettes/day), smoking spouse, smoking co-worker); diabetes history; age; thyroid history; residency; education; family history; iodized salt use; and nutrition (especially iodine status). Five patients were excluded from the study because of exclusion criteria: diabetes, protein deficiency, hepatic and renal dysfunction, thyroid active medication (e.g. amidarone, glucocorticoids, dopamine, propranolol, iodine, lithium, phenytoin, carbamazepine), systemic illnesses, or reporting thyroid disease. The final dataset included 255 study participants.

### Sample collection

Iodine, thiocyanate, nitrate and perchlorate levels were measured in 24-hour urine samples collected from residents of three cities in Turkey. Two low iodine cities (Isparta and Kayseri) and one iodine sufficient city (Istanbul) were chosen based on urinary iodine data from a previous pilot study [32]. The participants were recruited from Acibadem Hospital's clinics, including Acibadem International, Acibadem Bakırköy, Acibadem Kadıköy, Acibadem Kozyatağı in Istanbul, and from Suleyman Demirel University Hospital in Isparta, from University of Erciyes Hospital in Kayseri. All recruitment and data collection protocols were approved by the Medical Research Evaluation Committee of Acibadem University, and written informed consent for participation was obtained upon enrollment into the study.

Urine samples were collected between March and May in 2010 using standard plastic urine collection containers. The collection protocol started after the first morning urine on the first day was voided into the toilet (not collected); all subsequent urine was collected for the next 24 hours including the next day first morning urine. The volume of the 24-hr urine sample was measured, mixed and aliquots removed (5×5 ml) and stored frozen in 15 ml falcon tubes.

We chose non-lactating women because lactation complicates exposure assessment for these analytes: secretion into milk is a major pathway by which anions are cleared from a lactating woman's body. Perchlorate exposure is likely driven by diet, and thus non-lactating and non-pregnant women are likely to have the same exposure sources and exposure magnitudes as lactating and pregnant women (the most sensitive population) [33].

### Sample Analysis

Samples were analyzed for urine iodine concentration by inductively coupled plasma dynamic reaction cell mass spectrometry (ICP-DRC-MS), using an ELAN™ DRC II ICP-MS (Perkin Elmer Instruments, Shelton CT) using the method of Caldwell et al (2003, 2005) [34], [35]. Perchlorate, thiocyanate and nitrate were analyzed by triple-stage quadrupole ion chromatography-mass spectrometry (IC-MS/MS) using an Applied Biosystems 4000 IC-MS/MS system at the CDC (Centers for Disease Control and Prevention) in Atlanta, USA, using a slightly modified version of the method of Valentin-Blasini et al (2007) [36]. The accuracy of both methods was verified by blinded analysis of Standard Reference Material 3668 “Mercury, Perchlorate, and Iodide in Frozen Human Urine” from the National Institute of Standards and Technology [37]. Urinary creatinine concentrations were determined by a modified version of Jaffe method using Roche Modular Analyzer (Roche Diagnostics Corp., IN, USA) [38]. Creatinine excretion rates (g/24 h) were calculated by multiplying measured urinary creatinine concentration by the total volume of 24-hr urine collected. These calculated values were then compared with the estimated 24 hour creatinine excretion rates based on the sex, age, weight and height of the participants according to following formula [39]:
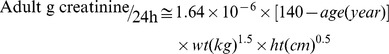



### Data Analysis

Concentrations of NIS inhibitors and iodine needed to be log-transformed to satisfy criteria of normality. Pearson correlation was used to evaluate bivariate relationships between analytes. Multivariate regression models were used to evaluate the relationship between analyte levels and variables that might impact exposure (e.g. age, BMI, and study site). Additionally, the iodine model included a categorical variable for iodized salt usage, and the thiocyanate model included categorical variables for self smoker, spouse smoker and co-worker smoker. All raw data from the study is freely available upon request.

## Results

The study participant characteristics are listed in Table 1. Average age was 35.5 years for the total study population, with Istanbul (mean  = 27.5±5.6) having significantly younger subjects compared with Kayseri (mean  = 36.5±9.7) and Isparta (mean  = 39.0±10.3). Mean body mass index (BMI) of all study participants was 26.1 kg/m^2^, which lies just above the overweight limit according to the World Health Organization [40]. Study participants in Istanbul (mean  = 21.9±3.2) had significantly lower BMI compared with Kayseri (mean  = 27.3±5.5) and Isparta (mean  = 27.2±4.8).

**Table 1:** Participant characteristics.

	Istanbul	Isparta	Kayseri
N (Total Participants)	58	98	99
**Age (%)**			
18–25	43	16	15
26–35	46	15	33
36–45	11	34	36
46–55	-	35	13
56–62	-	-	2
**BMI (%)**			
Underweight	5	3	3
Normal	83	30	38
Overweight	8	42	32
Obese	4	25	27
**Smoking Status (%)**			
Smoker	20	18	31
Non-smoker	80	82	69

BMI cutpoints (kg/m^2^): Underweight (<18.5); Normal (18.5–24.9); Overweight (25–30); Obese (>30). Smoker: total number of current smokers (light and heavy).

The 24-hr creatinine output was normally distributed with a mean of 1.11 g/24-hr. In order to evaluate the quality of urine collection, measured daily creatinine output was compared with estimated daily creatinine output. The average ratio of measured to estimated daily creatinine was 0.97, indicative of compliance of the study participants for collecting urine samples within the defined 24 hr study period. Furthermore, the 24 hr urinary volume was in the expected range (see Figure S3 in File S1), with a median of 1500 mL.

Median urinary iodine, perchlorate, nitrate and thiocyanate levels (µg/L) and doses (µg/kg/day) are listed in Table 2 for the three study locations. Urinary iodine levels tended to be higher in women using iodized salt (median  = 67.9 µg/L) compared with women not using iodized salt (median  = 47.8 µg/L), although this difference was not significant once we controlled for age, BMI and study site (β = 5.21 µg/L; p = 0.74). The overall median iodine excretion was 118.5 µg/day, with a log normal distribution as shown in Figure S1 in File S1. Urinary perchlorate levels and doses were also log normally distributed as shown in Table 2 and in Figure S1 in File S1. Furthermore, perchlorate exposure doses were lower in Isparta compared with Kayseri based on multivariate regression analysis that controlled for age and BMI (β = −0.048 µg/kg/day; p<0.0259). Urinary thiocyanate levels and doses were also log normally distributed as shown in Table 2 and in Figure S1 in File S1. Multivariate analysis found that smokers had significantly higher cyanide exposure compared with non-smokers (β = 1168 µg/L; p<0.0001), and that differences across study sites were not significant after controlling for smoking status, BMI and age. Urinary nitrate levels and doses were log normally distributed as shown in Table 2 and in Figure S1 in File S1. Urinary nitrate concentrations and doses were significantly lower in Isparta compared with Kayseri based on multivariate regression analysis that controlled for age and BMI (β = −13548 µg/L; p<0.05).

**Table 2:** Median urinary levels (µg/L) and doses (µg/kg/day) iodine, perchlorate, nitrate and thiocyanate in Isparta, Istanbul and Kayseri.

	Isparta		Istanbul		Kayseri	
	µg/L	µg/kg/day	µg/L	µg/kg/day	µg/L	µg/kg/day
Iodine	58.8	1.25	77.5	1.30	69.8	1.44
Perchlorate	5.01*	0.099*	5.63	0.096	7.72	0.167
Nitrate	21850	541*	48450	700	47450	1010
Thiocyanate	273	6.71	378	7.27	351	7.66

*Isparta lower than Kayseri, p<0.05.

**Figure 1:**
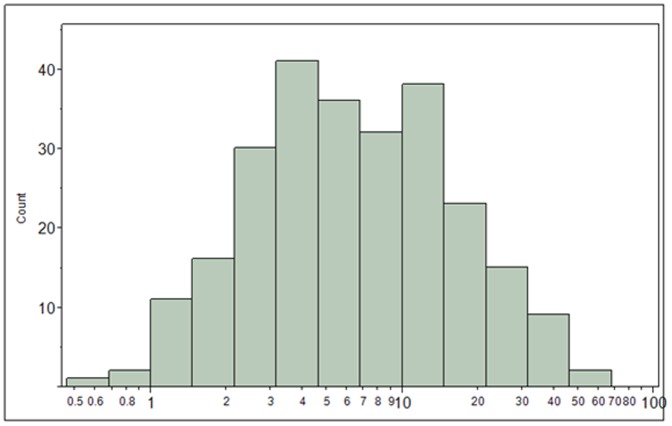
Distribution of urinary perchlorate excretion (µg/L) for the total study population.

Perchlorate exposure for the total study population is characterized graphically as a histogram of urinary perchlorate concentrations (µg/L) in Figure 1. Additionally, the 24-hr perchlorate excretion rate (µg/day) and estimated perchlorate dose (µg/kg/day) are shown in Figures S1 and S2 in File S1, respectively. The overall median perchlorate dose was 0.13 µg/kg/day.

**Figure 2:**
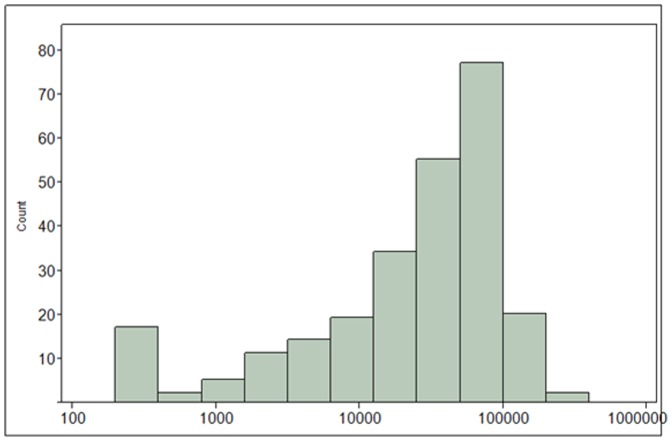
Distribution of urinary nitrate excretion (µg/L) for the total study population.

Nitrate exposure for the total study population is characterized graphically as a histogram of urinary nitrate concentrations (µg/L) in Figure 2. Additionally, the 24-hr nitrate excretion rate (µg/day) and estimated nitrate dose (µg/kg/day) are shown in Figures S1 and S2 in File S1, respectively. The overall median nitrate dose was 714 µg/kg/day.

**Figure 3:**
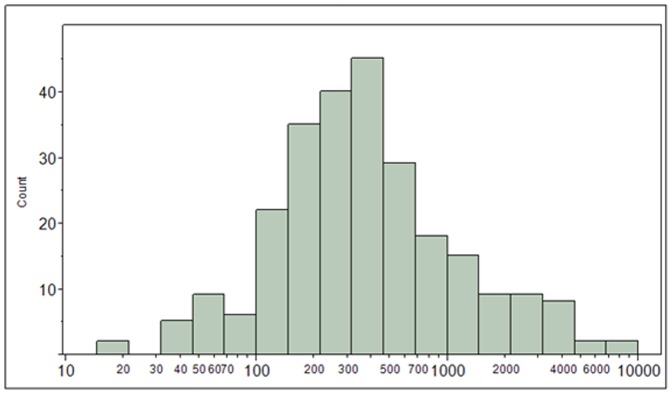
Distribution of urinary thiocyanate excretion (µg/L) for the total study population.

Cyanide exposures for the total study population are characterized graphically as a histogram of urinary thiocyanate concentrations (µg/L) in Figure 3. Median thiocyanate levels in all three groups of Turkish women were lower than median levels in US women in the National Health and Nutrition Examination Survey (NHANES) 2001−2002 (1260 µg/L) [41]. Additionally, the 24-hr thiocyanate excretion rate (µg/day) and estimated thiocyanate dose (µg/kg/day) are shown in Figures S1 and S2 in File S1, respectively. The overall median thiocyanate dose was 7.32 µg/kg/day.

**Figure 4:**
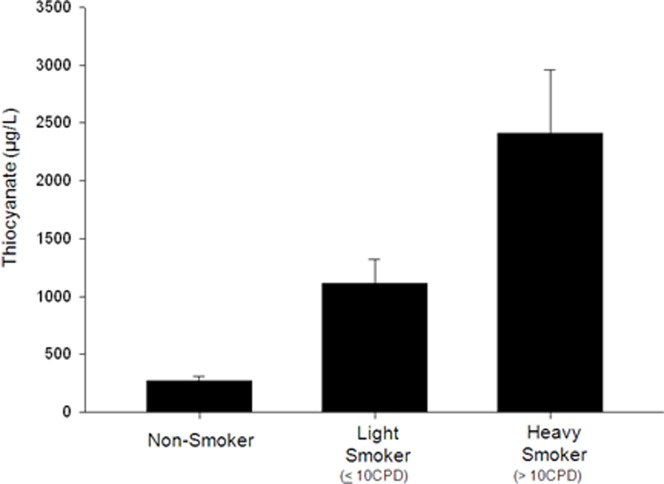
Urinary thiocyanate levels increase with increasing exposure to cyanide from tobacco smoke. (CPD: cigarettes per day).

The correlation of iodine and iodide uptake inhibitors (perchlorate, thiocyanate and nitrate) are illustrated in Figure 4 as a scatter plot matrix of 24-hr excretion rates. Correlation coefficients of log10-transformed data ranged from 0.09 to 0.38.

**Figure 5:**
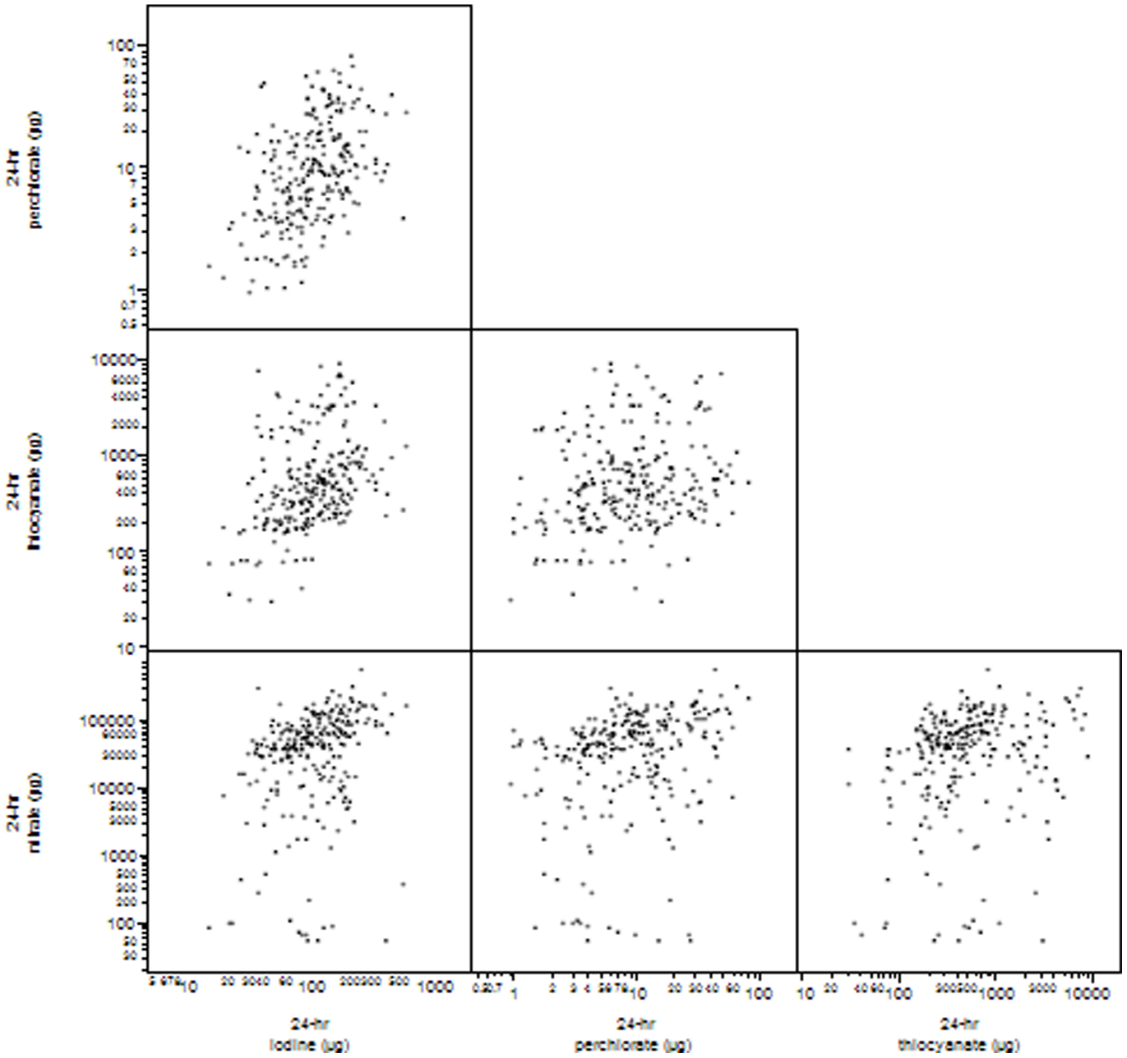
Scatter plot matrix showing correlations of urinary excretion of iodine and iodide uptake inhibitors (perchlorate, thiocyanate and nitrate).

Tobacco smoke exposure was associated with higher urinary thiocyanate levels as shown in Figure 5. Additional data on tobacco smoke exposure in the study population is shown in Figures S4a, S4b and S4c in File S1.

## Discussion

Perchlorate exposure has been associated with decreased thyroxine and increased thyroid stimulating hormone in women with lower iodine intakes in the U.S. population [11]. Further analyses find that low iodine intake coupled with concurrent exposure to multiple iodide uptake inhibitors (e.g. perchlorate, thiocyanate and nitrate) may decrease thyroid function [12], [31]. Turkey has a history of low iodine intake as well as potentially significant exposure to perchlorate and other iodide uptake inhibitors [16]. Therefore we designed this pilot study to investigate the prevalence of low iodine intake coupled with concurrent exposure to perchlorate, thiocyanate and nitrate. We found that the median urinary perchlorate concentration (6.4 µg/L) was more than twice as high as the median perchlorate concentration found in U.S. women (2.9 µg/L) [33]. Similarly, the median perchlorate dose across all Turkish sites (0.13 µg/kg/day) was 2.6 times higher than the median perchlorate dose found in U.S. women (0.051 µg/kg/day) [42]. Median perchlorate dose was below the U.S. EPA reference dose (0.7 µg/kg/day), but nine study participants had perchlorate doses higher than the U.S. EPA reference dose (see Figure S2 in File S1) [43]. Further study is needed to explore the potential impact of these perchlorate exposures.

The sources of perchlorate exposure in the study population are not known. Perchlorate enters the environment from both natural and anthropogenic sources and is stable in arid soils and water, leading to environmental persistence [29],[44]. Food and forage crops can uptake perchlorate from soil and irrigation water, leading to human exposure from consuming the food crops or from consuming milk produced by cattle fed perchlorate-contaminated forage crops [19], [28], [30], [45]. Thus, foods (leafy vegetables, milk products, and fruits) and drinking water may be significant contributors to perchlorate exposure in Turkey as well. Across the three cities studied, Isparta had lower perchlorate concentrations and doses compared with Kayseri (p<0.05; Table 2). Lower perchlorate exposure in Isparta could result from differences in locally grown food or local water disinfection practices [41],[46]. Additional data are needed to characterize perchlorate exposure sources in Turkey.

The recommended iodine intake for women of reproductive age is 150 µg/day [47]. The range of iodine excretion measured in 24 hr urine indicated that few of the study population consumed adequate levels of iodine (see Figure S1 in File S1). Populations are considered to have adequate iodine intake if the median urinary iodine levels are between 100−199 µg/L according to the WHO (World Health Organization) [48]. Our results (median urinary iodine  = 67.1 µg/L) agree with other studies that find that the Turkish population is moderately iodine deficient [32], . We found lower median levels of urinary iodine (67 µg/L) compared with a recent study by Erdogan et al (2007) that measured median iodine levels (107 µg/L) in morning urine samples of school-age children from 24 cities and from 7 regions in Turkey [16]. In the one city (Istanbul) that was sampled in both studies, Erdogan et al (2007) found twice the level of urinary iodine (154 µg/L compared to 77.5 µg/L). This difference in urinary iodine levels is attributable to the age of the study participants: children tend to have much higher urinary iodine levels compared with adults [34], [54]. In fact, urinary iodine data from NHANES consistently finds that women of reproductive age have about half the urinary iodine levels compared with children [54]. In contrast to NHANES data indicating adequate iodine intake in the US population, we found inadequate iodine intake (Table 2), suggesting ongoing iodine deficiency in all three cities studied.

The public health strategy to reduce iodine deficiency is salt iodization; therefore we expected higher iodine levels in urine collected from people who consume iodized salt. Urinary iodine levels were marginally higher in women using iodized salt (67.9 µg/L) compared with women not using iodized salt (47.8 µg/L), although this difference was not significant once we controlled for age, BMI and study site. According to the Turkey Demographic and Health Survey (2008) 15% of the households did not have iodized salt; furthermore, the availability of iodized salt differed by residence type and region [7]. In urban areas, only 10% of the household salt tested was not iodized, whereas this value goes up to 30% in rural areas. Despite the fact that 91% of the study participants reported using iodized salt in our study, the observed low levels of iodine intake indicate that additional efforts are needed to protect the Turkish population from iodine deficiency.

Istanbul participants were younger and of lower BMI than study participants from the other two locations. These demographic differences might affect the results. Previous reports indicate that people with higher BMI tend to excrete higher levels of perchlorate and other food-related anions [55]. Similarly, older U.S. adults tend to excrete more perchlorate than do younger U.S. adults, although the reason for this observation is not clear [55]. We controlled for differences in age and BMI between the three cities by using multivariate models. After adjusting for differences in age and BMI, urinary nitrate levels were lower in Isparta (21850 µg/L) compared with Kayseri (47450 µg/L, Table 2). The higher nitrate levels observed in Kayseri may result from higher levels of nitrate in local food and drinking water. Indeed the City of Kayseri Municipal Water and Sewer facility has reported nitrate levels as high as 49.25 mg/L [56], raising concerns about potential health effect [57]. Further work is needed to characterize nitrate exposure sources and health effects in Turkey.

Multivariate analysis found that smokers had significantly higher cyanide exposure compared with non-smokers (p<0.0001). The effect of smoking on the urinary thiocyanate levels is illustrated in Figure 4. Urinary thiocyanate levels increased with increasing cigarettes smoked per day (CPD), with heavy smokers (>10 CPD) having higher urinary thiocyanate levels (2410 µg/L) compared with light smokers (≤10 CPD, 1110 µg/L), who had higher urinary thiocyanate levels compared with non-smokers (268 µg/L). These higher thiocyanate levels are indicative of higher exposure to cyanide gas from tobacco smoke. Median thiocyanate levels in all three groups of Turkish women were lower than median levels in US women in the National Health and Nutrition Examination Survey (NHANES) 2001−2002 (1260 µg/L) [41], perhaps because Turkish women smoke fewer cigarettes compared with US women.

The scatter plot matrix illustrates correlations among analytes (Figure 5). Perchlorate, nitrate and iodine were more tightly correlated with each other than with thiocyanate, likely because of differences in exposure sources. Perchlorate, nitrate and iodine exposures are likely from the same sources (for example, dairy products tend to contain significant levels of both perchlorate and iodine, and leafy vegetables tend to contain significant levels of both perchlorate and nitrate). Conversely, tobacco smoke was the primary source of urinary thiocyanate as a metabolite of the cyanide in the tobacco smoke. We further explored second hand smoke exposure at home or at work as a potential source of thiocyanate, but did not find secondhand smoke categorization to be significantly related to increased urinary thiocyanate levels. Detailed distributions of tobacco smoke exposure results are shown in Figure S4a, Figure S4b and Figure S4c in File S1.

This pilot study provides novel data indicating that study participants had low iodine intake and high intake of some iodide uptake inhibitors compared with reference populations. However, the study also is weak in that it draws these conclusions based on a relatively small number of participants (N = 255) and possibly biased selection between study sites. Thus, our findings need to be confirmed in larger groups of participants, especially in pregnant and lactating women. While the study does use rigorous 24-hr urine collection, multiple 24-hr samples would have resulted in more precise exposure estimates. Additionally, the study would have been strengthened by full assessment of current thyroid function of study participants.

Individuals with lower ratios of iodine to iodide uptake inhibitors may be more prone to iodide uptake inhibition, with perchlorate, nitrate and thiocyanate possibly out-competing iodide for transport into the thyroid [1]. Chronically low levels of iodine relative to iodide uptake inhibitors could lead to decreased thyroid hormone production. Although our data only provide a 24-hr snap shot of the relative levels of iodide and iodide uptake inhibitors, it identifies lower levels of iodine and higher levels of perchlorate compared with U.S. reference data. Thus, iodide uptake may more likely be inhibited in this population compared to the U.S. population. For these reasons we aim to perform further studies to determine the sources of these contaminants, and to relate exposures to thyroid hormone levels.

## Supporting Information

File S1 **Contains: Figure S1**: Box and whisker plots of 24-hr urinary excretion rates calculated for iodine and iodine uptake inhibitors. **Figure S2**: Box and whisker plots of exposure doses calculated for perchlorate, nitrate, thiocyanate and iodine. **Figure S3**: Distribution of 24 hr urine volume for the total study population. **Figure S4a**: Prevalence and magnitude of active cigarette smoking in the total study population. Sm<10 =  light smoker, ≤10 cigarettes/day; Sm>10 =  heavy smoker, >10 cigarettes/day. **Figure S4b**: Prevalence and magnitude of spousal cigarette smoking in the total study population. Sm<10 =  light smoker, ≤10 cigarettes/day; Sm>10 =  light smoker, >10 cigarettes/day. **Figure S4c**: Prevalence and magnitude of exposure to coworkers smoking cigarettes. Sm<10 =  light smoker, ≤10 cigarettes/day; Sm>10 =  light smoker, >10 cigarettes/day.
(DOCX)

## References

[1] Andersson M, Karumbunathan V, Zimmermann MB (2012) Global Iodine Status in 2011 and Trends over the Past Decade. Journal of Nutrition 142: 744–750. 22378324 10.3945/jn.111.149393

[2] Stanbury JB, Ermans AE, Bourdoux P, Todd C, Oken E, et al (1998) Iodine-induced hyperthyroidism: occurrence and epidemiology. Thyroid 8: 83–100. 9492158 10.1089/thy.1998.8.83

[3] Hetzel BS (1983) Iodine deficiency disorders (IDD) and their eradication. Lancet 2: 1126–1129. 6138653 10.1016/s0140-6736(83)90636-0

[4] Laurberg P, Nohr SB, Pedersen KM, Hreidarsson AB, Andersen S, et al (2000) Thyroid disorders in mild iodine deficiency. Thyroid 10: 951–963. 11128722 10.1089/thy.2000.10.951

[5] Tonacchera M, Pinchera A, Dimida A, Ferrarini E, Agretti P, et al (2004) Relative potencies and additivity of perchlorate, thiocyanate, nitrate, and iodide on the inhibition of radioactive iodide uptake by the human sodium iodide symporter. Thyroid 14: 1012–1019. 15650353 10.1089/thy.2004.14.1012

[6] NRC (2005) Health Implications of Perchlorate Ingestion. Washington, DC: The National Academies Press.

[7] Hacettepe University Institute of Population Studies (2009) Turkey Demographic and Health Survey 2008. Ankara, Turkey: Hacettepe University Institute of Population Studies, Ministry of Health General Directorate of Mother and Child Health and Family Planning, T.R. Prime Ministry Undersecretary of State Planning Organization and TUBITAK. Available: http://www.hips.hacettepe.edu.tr/eng/tdhs08/TDHS-2008_Main_Report.pdf.

[8] Bilir N, Cakir B, Dagli E, Erguder T, Onder Z (2009) Tobacco Control in Turkey. Copenhagen, Denmark: World Health Organization Europe.

[9] Knudsen N, Bulow I, Laurberg P, Ovesen L, Perrild H, et al (2002) Association of tobacco smoking with goiter in a low-iodine-intake area. Archives of Internal Medicine 162: 439–443. 11863477 10.1001/archinte.162.4.439

[10] Wang H, Sekine M, Yokokawa H, Hamanishi S, Sayama M, et al (2001) Serum Thiocyanate Concentration as an Indicator of Smoking in Relation to Deaths from Cancer. Environmental Health and Preventive Medicine 6: 88–91. 21432242 10.1007/BF02897951 PMC2723241

[11] Blount BC, Pirkle JL, Osterloh JD, Valentin-Blasini L, Caldwell KL (2006) Urinary perchlorate and thyroid hormone levels in adolescent and adult men and women living in the United States. Environmental Health Perspectives 114: 1865–1871. 17185277 10.1289/ehp.9466 PMC1764147

[12] Steinmaus C, Miller MD, Howd R (2007) Impact of smoking and thiocyanate on perchlorate and thyroid hormone associations in the 2001−2002 National Health and Nutrition Examination Survey. Environmental Health Perspectives 115: 1333–1338. 17805424 10.1289/ehp.10300 PMC1964908

[13] Kutlu AO, Kara C (2012) Iodine deficiency in pregnant women in the apparently iodine-sufficient capital city of Turkey. Clinical Endocrinology 77: 615–620. 22587848 10.1111/j.1365-2265.2012.04440.x

[14] Gurkan R, Bicer N, Ozkan MH, Akcay M (2004) Determination of trace amounts of iodide by an inhibition kinetic spectrophotometric method. Turkish Journal of Chemistry 28: 181–191.

[15] Kut A, Gursoy A, Senbayram S, Bayraktar N, Budakoglu, II, et al (2010) Iodine intake is still inadequate among pregnant women eight years after mandatory iodination of salt in Turkey. Journal of Endocrinological Investigation 33: 461–464. 20061785 10.1007/BF03346625

[16] Erdogan MF, Agbaht K, Altunsu T, Ozbas S, Yucesan F, et al (2009) Current iodine status In Turkey. Journal of Endocrinological Investigation 32: 617–622. 19564718 10.1007/BF03346519

[17] Erdogan MF (2007) Iyot profilaksisi sonrası nerdeyiz? 2007 Türkiye tarama sonuçları. Endokrinolojide Diyalog 4: 211–215.

[18] Kirk AB, Martinelango PK, Tian K, Dutta A, Smith EE, et al (2005) Perchlorate and iodide in dairy and breast milk. Environmental Science & Technology 39: 2011–2017. 15871231 10.1021/es048118t

[19] Blount BC, Ozpinar A, Alwis KU, Caudill SP, Gillespie JR (2008) Perchlorate, Nitrate, Thiocyanate, and Iodide Levels in Chicken Feed, Water, and Eggs from Three Farms. Journal of Agricultural and Food Chemistry 56: 10709–10715. 18959414 10.1021/jf8018326

[20] Greer MA, Goodman G, Pleus RC, Greer SE (2002) Health effects assessment for environmental perchlorate contamination: The dose response for inhibition of thyroidal radioiodine uptake in humans. Environmental Health Perspectives 110: 927–937. 12204829 10.1289/ehp.02110927 PMC1240994

[21] Urbansky ET (2002) Perchlorate as an environmental contaminant. Environmental Science and Pollution Research 9: 187–192. 12094532 10.1007/BF02987487

[22] Guruge KS, Wu Q, Kannan K (2011) Occurrence and exposure assessment of perchlorate, iodide and nitrate ions from dairy milk and water in Japan and Sri Lanka. Journal of Environmental Monitoring 13: 2312–2320. 21738937 10.1039/c1em10327j

[23] Kucharzyk KH, Crawford RL, Cosens B, Hess TF (2009) Development of drinking water standards for perchlorate in the United States. Journal of Environmental Management 91: 303–310. 19850401 10.1016/j.jenvman.2009.09.023

[24] McLaughlin CL, Blake S, Hall T, Harman M, Kanda R, et al (2011) Perchlorate in raw and drinking water sources in England and Wales. Water and Environment Journal 25: 456–465.

[25] Her N, Kim J, Yoon Y (2010) Perchlorate in dairy milk and milk-based powdered infant formula in South Korea. Chemosphere 81: 732–737. 20692011 10.1016/j.chemosphere.2010.07.031

[26] Oh S-H, Lee J-W, Mandy P, Oh J-E (2011) Analysis and Exposure Assessment of Perchlorate in Korean Dairy Products with LC-MS/MS. Environ Health Toxicol 26. 10.5620/eht.2011.26.e2011011 PMC3214986 22125772

[27] Asami M, Kosaka K, Yoshida N (2009) Occurrence of Chlorate and Perchlorate in Bottled Beverages in Japan. Journal of Health Science 55: 549–553.

[28] Ha W, Suarez DL, Lesch SM (2011) Perchlorate Uptake in Spinach As Related to Perchlorate, Nitrate, And Chloride Concentrations in Irrigation Water. Environmental Science & Technology. 10.1021/es2010094 21939238

[29] Sanchez CA, Krieger RI, Khandaker NR, Valentin-Blasini L, Blount BC (2006) Potential perchlorate exposure from Citrus sp irrigated with contaminated water. Analytica Chimica Acta 567: 33–38. 17723376 10.1016/j.aca.2006.02.013

[30] Yang M, Her N (2011) Perchlorate in Soybean Sprouts (Glycine max L. Merr.), Water Dropwort (Oenanthe stolonifera DC.), and Lotus (Nelumbo nucifera Gaertn.) Root in South Korea. Journal of Agricultural and Food Chemistry 59: 7490–7495. 21627073 10.1021/jf2009638

[31] Gatseva P, Vladeva S, Argirova M (2007) Evaluation of endemic goiter prevalence in Bulgarian schoolchildren - Results from national strategies for prevention and control of iodine-deficiency disorders. Biological Trace Element Research 116: 273–278. 17709907 10.1007/BF02698011

[32] Erdogan G EM, Ustundag M, Haznedaroglu D, Kose R, Genc Y (2003) 2002 yili 20 bölge Türkiye iyot durumu monitorizasyon projesi kesin raporu [Final report of the iodine status monitoring project in 20 regions in Turkey, 2002]. Ankara: Ankara University, Medical School, Department of Endocrinology and Metabolism.

[33] Blount BC, Valentin-Blasini L, Osterloh JD, Mauldin JP, Pirkle JL (2007) Perchlorate exposure of the US Population, 2001−2002. J Expo Sci Environ Epidemiol 17: 400–407. 17051137 10.1038/sj.jes.7500535

[34] Caldwell KL, Jones R, Hollowell JG (2005) Urinary iodine concentration: United States National Health and Nutrition Examination Survey 2001−2002. Thyroid 15: 692–699. 16053386 10.1089/thy.2005.15.692

[35] Caldwell KL, Maxwell CB, Makhmudov A, Pino S, Braverman LE, et al (2003) Use of inductively coupled plasma mass spectrometry to measure urinary iodine in NHANES 2000: Comparison with previous method. Clinical Chemistry 49: 1019–1021. 12766019 10.1373/49.6.1019

[36] Valentin-Blasini L, Blount BC, Delinsky A (2007) Quantification of iodide and sodium-iodide symporter inhibitors in human urine using ion chromatography tandem mass spectrometry. Journal of Chromatography A 1155: 40–46. 17466997 10.1016/j.chroma.2007.04.014

[37] Yu LL, Jarrett JM, Davis WC, Kilpatrick EL, Oflaz R, et al (2012) Characterization of perchlorate in a new frozen human urine standard reference material. Anal Bioanal Chem 404: 1877–1886. 22850897 10.1007/s00216-012-6263-5 PMC4542061

[38] Junge W, Wilke B, Halabi A, Klein G (2004) Determination of reference intervals for serum creatinine, creatinine excretion and creatinine clearance with an enzymatic and a modified Jaffe method. Clinica Chimica Acta 344: 137–148. 10.1016/j.cccn.2004.02.007 15149882

[39] Mage DT, Allen RH, Gondy G, Smith W, Barr DB, et al (2004) Estimating pesticide dose from urinary pesticide concentration data by creatinine correction in the Third National Health and Nutrition Examination Survey (NHANES-III). Journal of Exposure Analysis and Environmental Epidemiology 14: 457–465. 15367927 10.1038/sj.jea.7500343

[40] WHO Expert Committee on Physical Status: the Use and Interpretation of Anthropometry (1993: Geneva Switzerland), World Health Organization. (1995) Physical status: The use and interpretation of anthropometry, report of a WHO expert committee. Geneva: World Health Organization. 8594834

[41] Blount BC, Alwis KU, Jain RB, Solomon BL, Morrow JC, et al (2010) Perchlorate, Nitrate, and Iodide Intake through Tap Water. Environmental Science & Technology 44: 9564–9570. 21090606 10.1021/es1025195

[42] Huber DR, Blount BC, Mage DT, Letkiewicz FJ, Kumar A, et al (2011) Estimating perchlorate exposure from food and tap water based on US biomonitoring and occurrence data. Journal of Exposure Science and Environmental Epidemiology 21: 395–407. 20571527 10.1038/jes.2010.31

[43] U.S. Environmental Protection Agency (2008) Interim Drinking Water Health Advisory for Perchlorate. Washington, DC: U.S. EPA. EPA/822/R-08/025. Available: http://www.epa.gov/safewater/contaminants/unregulated/pdfs/healthadvisory_perchlorate_interim.pdf.

[44] Brandhuber P, Clark S, Morley K (2009) A review of perchlorate occurrence in public drinking water systems. Journal: American Water Works Association 101: 63–73.

[45] Shi Y, Zhang P, Wang Y, Shi J, Cai Y, et al (2007) Perchlorate in sewage sludge, rice, bottled water and milk collected from different areas in China. Environment International 33: 955–962. 17604836 10.1016/j.envint.2007.05.007

[46] Zewdie T, Smith CM, Hutcheson M, West CR (2010) Basis of the Massachusetts Reference Dose and Drinking Water Standard for Perchlorate. Environmental Health Perspectives 118: 42–48. 20056583 10.1289/ehp.0900635 PMC2831965

[47] Becker DV, Braverman LE, Delange F, Dunn JT, Franklyn JA, et al (2006) Iodine supplementation for pregnancy and lactation - United States and Canada: Recommendations of the American Thyroid Association. Thyroid 16: 949–951. 17042677 10.1089/thy.2006.16.949

[48] World Health Organization (2007) Assessment of iodine deficiency disorders and monitoring their elimination: A guide for programme managers. 3 ed.

[49] Andersson M, Takkouche B, Egli I, Allen HE, de Benoist B (2005) Current global iodine status and progress over the last decade towards the elimination of iodine deficiency. Bulletin of the World Health Organization 83: 518–525. 16175826 PMC2626287

[50] Gultepe M, Ozcan O, Ipcioglu OM (2005) Assessment of iodine intake in mildly iodine-deficient pregnant women by a new automated kinetic urinary iodine determination method. Clin Chem Lab Med 43: 280–284. 15843231 10.1515/CCLM.2005.047

[51] Kurtoglu S, Akcakus M, Kocaoglu C, Gunes T, Karakucuk I, et al (2004) Iodine deficiency in pregnant women and in their neonates in the central Anatolian region (Kayseri) of Turkey. Turk J Pediatr 46: 11–15. 15074368

[52] Cetin H, Kisioglu AN, Gursoy A, Bilaloglu E, Ayata A (2006) Iodine deficiency and goiter prevalence in Turkey after mandatory iodization. J Endocrinol Invest 29: 714–718. 17033260 10.1007/BF03344181

[53] Gur E, Ercan O, Can G, Akkus S, Guzeloz S, et al (2003) Prevalence and risk factors of iodine deficiency among schoolchildren. J Trop Pediatr 49: 168–171. 12848208 10.1093/tropej/49.3.168

[54] Caldwell KL, Makhmudov A, Ely E, Jones RL, Wang RY (2011) Iodine Status of the U.S. Population, National Health and Nutrition Examination Survey, 2005−2006 and 2007−2008. Thyroid 21: 419–427. 21323596 10.1089/thy.2010.0077

[55] Lau FK, deCastro BR, Mills-Herring L, Tao L, Valentin-Blasini L, et al (2013) Urinary perchlorate as a measure of dietary and drinking water exposure in a representative sample of the United States population 2001−2008. Journal of Exposure Science and Environmental Epidemiology 23: 207–214. 23188482 10.1038/jes.2012.108

[56] KASKI (2012) Icme-Kullanma Suyu Analiz Raporu (City of Kayseri tap water analysis report) July 2012. Kayseri, Turkey: Kayseri Buyuksehir Belediyesi Su ve Kanalizasyon Idaresi Genel Mudurlugu. Available: http://www.kaski.gov.tr/images/dosyalar/20120803164610_0.pdf.

[57] van Grinsven HJ, Ward MH, Benjamin N, de Kok TM (2006) Does the evidence about health risks associated with nitrate ingestion warrant an increase of the nitrate standard for drinking water? Environ Health 5: 26. 16989661 10.1186/1476-069X-5-26 PMC1586190

