# Supplementary material for: Iodine Status in Turkish Populations and Exposure to Iodide Uptake Inhibitors
Source: PLoS One. 2014 Feb 5;9(2):e88206. doi: 10.1371/journal.pone.0088206 (PMC3914924; doi:10.1371/journal.pone.0088206)
Supplement: File S1 — Contains: Figure S1: Box and whisker plots of 24-hr urinary excretion rates calculated for iodine and iodine uptake inhibitors. Figure S2: Box and whisker plots of exposure doses calculated for perchlorate, nitrate, thiocyanate and iodine. Figure S3: Distribution of 24 hr urine volume for the total study population. Figure S4a: Prevalence and magnitude of active cigarette smoking in the total study population. Sm<10 = light smoker, ≤10 cigarettes/day; Sm>10 = heavy smoker, >10 cigarettes/day. Figure S4b: Prevalence and magnitude of spousal cigarette smoking in the total study population. Sm<10 = light smoker, ≤10 cigarettes/day; Sm>10 = light smoker, >10 cigarettes/day. Figure S4c: Prevalence and magnitude of exposure to coworkers smoking cigarettes. Sm<10 = light smoker, ≤10 cigarettes/day; Sm>10 = light smoker, >10 cigarettes/day. (DOCX) [file pone.0088206.s001.docx]

**Supporting Information**


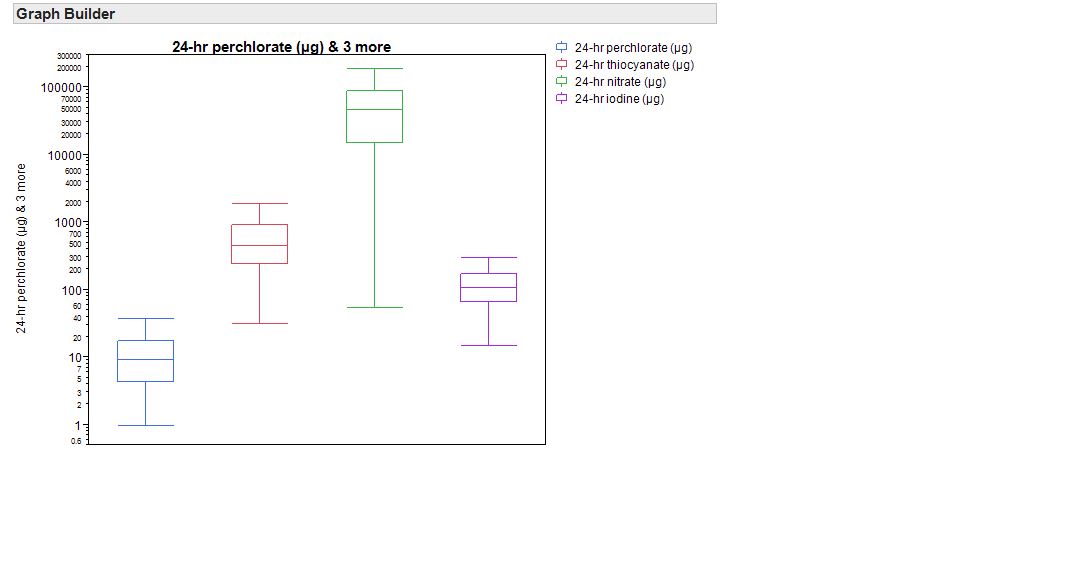
One of the strengths of our study was that we collected 24 hr urines and thus integrated exposure over an entire 24 hr time. Conversely, a spot urine normally only reflects a few hours of exposure, and thus can be imprecise. As a result our data more precisely reflect the time averaged exposure of study participants to the analytes period. The 24 hr urinary output of iodine indicates that the population is mildly iodine deficient. We also display the relative levels of excretion of iodide uptake inhibitors in Figure S1 for comparison with iodine levels.

**Figure S1:** Box and whisker plots of 24-hr urinary excretion rates calculated for iodine and iodine uptake inhibitors.

Exposure biomarkers are often measurable at levels below levels known to impact health. To gain toxicological perspective on the measured levels of perchlorate, we calculated daily dose based on perchlorate concentration, body weight and 24 urine volume (Figure S2). For comparison sake we also display the doses of iodine, nitrate and thiocyanate in Figure S2 for comparison with perchlorate doses.


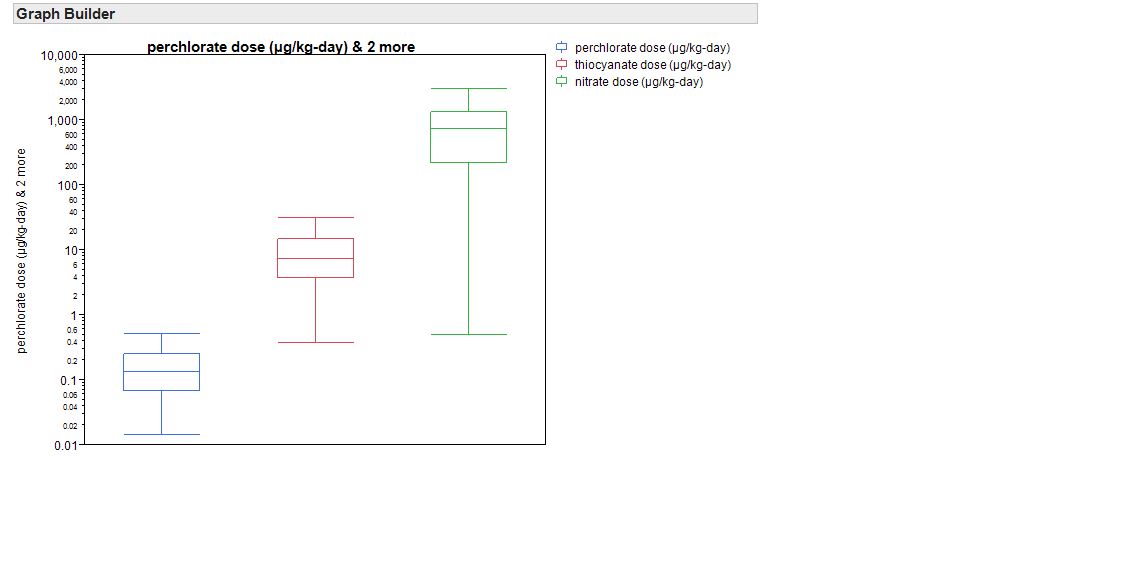


**Figure S2:** Box and whisker plots of exposure doses calculated for perchlorate, nitrate, and thiocyanate.

We validated the completeness of 24-hr urine by comparing measured creatinine output with expected creatinine output. In order to evaluate the quality of urine collection, measured daily creatinine output was compared with estimated daily creatinine output. The average ratio of measured to estimated daily creatinine was 0.97. This indicates the compliance of the study participants for collecting urine samples within the defined 24h study period. Additionally, the volume of urine collected for each study participant was in the expected range. The distribution of urine volumes for 24-hour urine samples are given in Figure S3. The median urine volume was 1500 ml.

**Figure S3.** Distribution of 24 hr urine volume for the total study population.


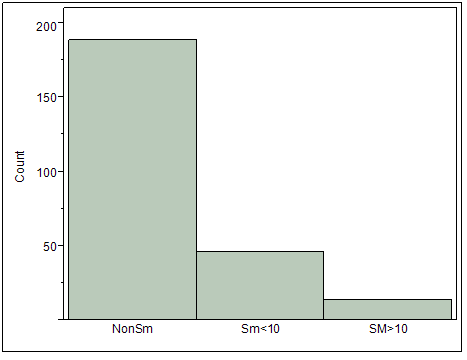


Figure S4a: Prevalence and magnitude of active cigarette smoking in the total study population. Sm<10 = light smoker, <10 cigarettes/day; Sm>10 = heavy smoker, >10 cigarettes/day.


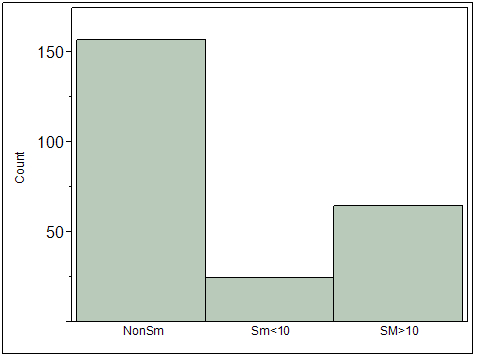


Figure S4b: Prevalence and magnitude of spousal cigarette smoking in the total study population. Sm<10 = light smoker, <10 cigarettes/day; Sm>10 = light smoker, >10 cigarettes/day.


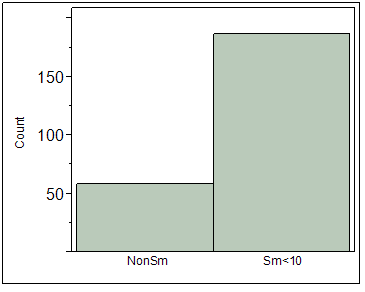


Figure S4c: Prevalence and magnitude of exposure to coworkers smoking cigarettes. Sm<10 = light smoker, <10 cigarettes/day; Sm>10 = light smoker, >10 cigarettes/day.
